# When Is a Species Declining? Optimizing Survey Effort to Detect Population Changes in Reptiles

**DOI:** 10.1371/journal.pone.0043387

**Published:** 2012-08-22

**Authors:** David Sewell, Gurutzeta Guillera-Arroita, Richard A. Griffiths, Trevor J. C. Beebee

**Affiliations:** 1 Durrell Institute of Conservation and Ecology, School of Anthropology and Conservation, University of Kent, Canterbury, Kent, United Kingdom; 2 School of Life Sciences, University of Sussex, Falmer, Brighton, United Kingdom; 3 National Centre for Statistical Ecology, School of Mathematics, Statistics and Actuarial Science, University of Kent, Canterbury, Kent, United Kingdom; University of Western Ontario, Canada

## Abstract

Biodiversity monitoring programs need to be designed so that population changes can be detected reliably. This can be problematical for species that are cryptic and have imperfect detection. We used occupancy modeling and power analysis to optimize the survey design for reptile monitoring programs in the UK. Surveys were carried out six times a year in 2009–2010 at multiple sites. Four out of the six species – grass snake, adder, common lizard, slow-worm –were encountered during every survey from March-September. The exceptions were the two rarest species ­– sand lizard and smooth snake – which were not encountered in July 2009 and March 2010 respectively. The most frequently encountered and most easily detected species was the slow-worm. For the four widespread reptile species in the UK, three to four survey visits that used a combination of directed transect walks and artificial cover objects resulted in 95% certainty that a species would be detected if present. Using artificial cover objects was an effective detection method for most species, considerably increased the detection rate of some, and reduced misidentifications. To achieve an 85% power to detect a decline in any of the four widespread species when the true decline is 15%, three surveys at a total of 886 sampling sites, or four surveys at a total of 688 sites would be required. The sampling effort needed reduces to 212 sites surveyed three times, or 167 sites surveyed four times, if the target is to detect a true decline of 30% with the same power. The results obtained can be used to refine reptile survey protocols in the UK and elsewhere. On a wider scale, the occupancy study design approach can be used to optimize survey effort and help set targets for conservation outcomes for regional or national biodiversity assessments.

## Introduction

There is widespread evidence of worldwide declines in populations of vertebrates [1] including fish [2], [3], amphibians [4], [5], [6], reptiles [7], [8], birds [9], [10], [11] and mammals [12]. Declines have been attributed to a number of causes including habitat loss or change [7]; disease [13]; pollution [2] and climate change [14], [15], [16]. Action to address these declines requires sound scientific data on population trends at different scales. At the individual population level, long-term monitoring can provide useful data on the nature of population fluctuations and drivers of population change [17]. However, what may be more useful for conservation purposes are data on changes in the number of populations at the wider landscape level [18]. Such approaches present challenges in terms of logistics and expertise, particularly for cryptic species that are difficult to detect and identify. This raises the issue of how much survey effort is required to reliably identify population changes.

A National Amphibian and Reptile Recording Scheme (NARRS) was instituted in Britain in 2007, with a view to assessing future status changes of the herpetofauna of the UK (United Kingdom), including the six native reptile species, slow-worm *Anguis fragilis* Linnaeus 1758, common or viviparous lizard *Zootoca vivipera* Jacquin 1787, sand lizard *Lacerta agilis* Linnaeus, 1758, adder *Vipera berus* Linnaeus 1758, grass snake *Natrix natrix* Linnaeus 1758 and smooth snake *Coronella austriaca* Laurenti 1768. NARRS primarily uses trained volunteers who carry out presence-absence surveys using a standard protocol. In this study, we applied an occupancy modeling technique that accounts for imperfect detection to the current NARRS survey protocol for reptiles. This modeling framework is based on the patterns of detection and non-detection of species at a range of sites over multiple visits, and estimates both site occupancy and detection probability simultaneously [19].

Once the detection probability of any particular species is estimated it is possible to determine the number of survey visits required at an occupied site for the species to be detected to a given level of certainty [20]–[23]; note that a similar assessment can be performed without conditioning on species presence [24]. Survey effort can be allocated into number of sites and number of survey visits in different ways. For a given combination of occupancy and detection probabilities, there is an optimal level of replication in terms of estimator precision [25], [26], [27]. Thus, if sufficient surveyors are available to carry out 1000 surveys, is it better to survey 250 sites four times or 100 sites ten times? Such decisions are important for optimizing the deployment of survey effort. Equally, it is essential to design the survey so that biologically significant changes in species occupancy over time can be detected reliably.

In this study we used an occupancy modeling approach on all six native reptile species in the UK to determine (1) occupancy and detectability of all the species across a range of sites; (2) the optimal number of survey visits to carry out per site; and (3) the required sampling effort to detect population declines at different power levels for the four commoner species.

## Materials and Methods

### Study Areas and Field Work

In 2009 29 sites were surveyed for reptiles on six occasions, once each in March, April, May, June, July and September. All chosen sites were in southeast England as this was the only part of the United Kingdom where all six native reptile species occurred. In 2010 the same sites were again surveyed six times and additional sites were incorporated to bring the total sample size to 45 (see Fig. S1). These were from a wider geographical area, embracing Wales to the west, East Anglia to the east and Yorkshire to the north. The 45 site data set was thus more representative of the UK as a whole but did not increase coverage of the two rarest species, both of which have a highly restricted UK distribution.

Surveys were carried out by a mixture of volunteer and professional surveyors. All volunteers had received initial training on survey methods and species identification. Surveys consisted of recording the number of detections of each species using two methods: (i) a search under natural and artificial cover objects and (ii) visual searches to find basking or active animals along directed transect routes. The aim was to have 30 artificial cover objects (ACOs) per site but the actual number and type of ACO varied between sites as would be expected in a volunteer programme. Two main types of ACO were used: corrugated tin and felts. The size of individual ACOs was within the range 0.5 m×0.5 m to 1.0 m×2.0 m. Pre-existing refugia were also surveyed. These were non-natural items that had been laid or discarded by people other than the surveyor and included rubble, car tires, doors and sheets of various building materials. By definition a surveyor had no control over the number or type of pre-existing refugia found on a site. The length of the survey route followed for the visual searches varied according to the topography and size of the site. The only limitation imposed was that total time spent on a single site survey was not permitted to exceed three hours, in accordance with NARRS guidelines.

The term ‘encounter’ is used here to denote survey occasions on which a species was detected, not the number of individuals. As well as individual(s) an encounter could consist of any matter offering evidence that a species was occupying a site, for example a sloughed skin identifiable to species level. Sloughs found under ACOs were immediately removed to ensure that any further sloughs found on subsequent surveys were fresh.

Data on site-specific factors thought to be potentially relevant for species site occupancy were collected, many based on those collected as part of the NARRS survey protocol, and others based on previous survey programs [28]. These included information on site area and compass orientation, soil type, connectivity and human impact. Site area was the part of a site actually surveyed, measured in hectares. The percentages of area within each site either level or south facing were recorded. Soil type was determined from visible substrate and flora according to three categories: acid (sandy soils); neutral (mainly clay soils), and alkaline (mainly chalk soils). The level of connectivity of the site to other suitable reptile habitat was recorded on a four point scale: 1– completely isolated; 2– isolated by sub-optimal habitat; 3– linked by corridors of good habitat and 4– part of a larger area of good habitat. Human impact, a term encompassing anthropogenic activity, from forestry and agricultural uses on the site through to the recreational impacts of cyclists, dog walkers and ramblers, was recorded on a five point scale: 1– heavily used; 2– heavily used during parts of the day only; 3– moderate use; 4– low people impact, site probably used by people only at weekends, and 5– little to no human impact.

Data on survey-specific factors thought potentially to affect species detection probabilities were also collected. These included information on weather conditions, survey duration, number of ACOs examined and observer skills. Maximum and minimum air and soil temperatures were recorded for each survey. The mean was taken for both the air and soil readings and used in the analysis. Cloud cover was estimated as the percentage cover of the visible sky. Previous studies have shown that both temperature and cloud cover influence the capture rate of snakes [28]. Duration of the survey was timed in minutes from start to finish. Although a specific number of ACOs were allocated to a site at the start of the survey season, the actual quantity examined varied among survey visits. ACOs were occasionally trampled and destroyed by livestock or vandalism and during the summer months could become difficult to locate due to vegetation growth. The skill of the most experienced observer carrying out each survey was assessed by the lead surveyor as a survey-specific variable to allow for the improvement in survey ability of individuals as the programme progressed. Four experience categories were designated: ‘1’ trained but with little practical experience; ‘2’ reasonable level of experience or a good knowledge of the site being surveyed but not both; ‘3’ good practical experience and knowledge of the site being surveyed; ‘4’ reserved for a few surveyors of proven high ability and experience.

### Data Analysis

A detection history was constructed for each species, assigning a ‘1’ to those survey visits in which the species was detected at least once using any of the detection methods and a ‘0’ otherwise. Data were analyzed using an occupancy modeling technique that explicitly accounts for imperfect detection [19]. This technique relies on replicated detection/non-detection surveys to model the detection process at occupied sites and produces estimates for both occupancy and detection probability. The analysis was carried out in program PRESENCE version 4.0 (freely available from http://www.mbr-pwrc.usgs.gov/software/presence.html). Data were analyzed as three different sets: 29 sites from 2009, 45 sites from 2010 and a reduced 2010 data set of 29 sites to provide a direct comparison with the results from the previous year. We incorporated into the models the collected site and survey-specific covariates on occupancy (*ψ*) and detection probability (*p*) respectively, through a logit link function. Covariates were incorporated individually and therefore the total number of models tested for each species in each data set was 15. The exception to this general rule was when a species was found at all study sites. Models incorporating site covariates were not considered in this situation, reducing the number of models tested to 8. We used AIC to rank the candidate models for each species and data set, discarding those that failed to converge. Overall estimates of occupancy and detection probability were obtained for each of the models in the set by averaging the individual site/survey estimates and corresponding standard errors were computed from the variance-covariance matrix of the logistic regression coefficients using the delta method. These overall estimates were then used to obtain model-averaged estimates [29], [30] based on all the models in the set.

### Survey Design Recommendations

Survey design recommendations were derived based on the estimates obtained for occupancy and detectability. Where survey-specific detection probabilities resulted from the analysis, the mean of these was used for the calculation. We started by exploring the number of survey visits (*K*) required to determine species presence at an occupied site with a given probability. The probability of detecting the species at an occupied site in at least one of the visits is p* = 1–(1–*p*)*^K^*, where *p* is the detection probability on any one visit. From this expression the number of visits (*K*) required to achieve a given desired p* (0.80, 0.90 or 0.95) was derived as [20]–[23]:
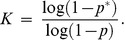
(1)


We then assessed what would be a suitable design for future surveys to detect an occupancy decline in any of the four commoner species (i.e. slow-worm, common lizard, adder and grass snake). We based this on the estimates of occupancy and detection probability from the 2010 45-site data set and assumed a standard sampling design in which *S* sites are sampled *K* repeated times. We first determined for each species the optimal amount of replication *K* for the estimated occupancy and detectability levels. This was chosen as the replication that, for a fixed total survey effort *E* = *KS*, minimizes the asymptotic variance of the occupancy estimator [25], [27] given by:
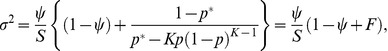
(2) where the term *F* inflates the variance of the occupancy estimator with respect to the variance of a binomial proportion, and tends to zero as the probability of missing the species at occupied sites 1-p* tends to zero. Based on this amount of replication, we determined the number of sampling sites needed to achieve a given power to detect (1– β) a decline in occupancy between two surveys given an actual proportional decline (effect size) *R* as follows [31]

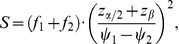
(3) where *ψ*1 is the initial occupancy, *ψ*2 = *ψ*1 (1– R ) is the resulting occupancy after a proportional change R (>0 for a decline, <0 for an increase), *f*1 = *ψ*1 (1–*ψ*1+F ), *f*2 = *ψ*2 (1–*ψ*2+F ), and zi is the upper 100α/2-percentage point for the standard normal distribution. This expression is based on large-sample approximations and assumes independence in the occupancy status of each site between the two samples. We assessed three effect sizes (R = 30%, 15% and 10%) using a significance level α = 0.05. As an initial step we explored the study design for each species individually, and then considered the results in light of the multispecies nature of the survey. We assumed that detection probability and the number of replicate surveys were the same in the two sampling occasions. Finally, since eq 3 is an approximate expression, we used simulations to verify the actual performance of the identified designs. In each simulation two detection histories (with *ψ*1 and *ψ*2) were generated and analyzed. This was repeated 5000 times for each scenario and power was computed as the proportion of simulations in which a significant decline was detected using a Wald test [32]. R code that implements eq3 as well as power simulations can be found in [31].

## Results

### Site-specific Variables

The 29- site sample comprised 11 on acid, 9 on neutral and 9 on alkaline soil. The 45- site data set consisted of 16 on acid, 17 on neutral and 12 on alkaline soil. The average area (and associated S.D.) in the ‘29 sites’ sample was 12.5 (16.40) hectares, but the additions in the ‘45 sites’ sample reduced both mean area and S.D. to 9.8 (13.36) hectares. The mean percentage of each site that was south facing was reasonably constant between the data sets at 31.0 (33.47) for the 29 sites compared to 29.3 (35.22) for the 45 sites. The percentage of each site that was level was also similar at 42.0 (38.35) for the 29 sites and 48.0 (40.37) for 45 sites. The wide standard deviations indicate high variations within each data set.

### Survey-specific Variables

Most of the survey-specific variables were similar between year and data set. For example, mean air temperature was 17.4°C (3.41) in the 2009 data set, 17.4°C (4.68) in the 29- sites 2010 data set and 17.1°C (4.67) for the 2010 45 sites data set. There was a significant correlation between mean air and soil temperatures in all three data sets (Pearson’s r between 0.74 and 0.57, *P*<0.01).

### Survey Results

All six species were encountered in every survey round (Table S1), with two exceptions. In 2009 no sand lizards were found in July, whilst in 2010 there were no records of smooth snakes in March. The most frequently encountered species was the slow-worm, encountered at all sites in both years in the 29 site data set but absent from some sites in the larger 45 site data set. Conversely sand lizards and smooth snakes were the two least frequently encountered species, with the number of encounters constant between years. In the 29 sites data set there were a total of 15 sand lizard encounters in 2009 and 16 in 2010, whilst the corresponding figures for smooth snakes were 27 and 26 respectively. For these two species, which have restricted UK distributions, the larger 45 sites data set did not include any additional sites within their range.

The most effective method to obtain an encounter varied between species. For slow-worms, smooth snakes and grass snakes the inspection of ACOs was far more likely to result in an encounter with the species than directed transects (Table S2). Conversely, for sand lizards, transects were clearly more effective than ACOs. For the remaining two species both detection methods were effective in yielding detections (Table S2).

### Occupancy Modeling

The estimated occupancy and detection probabilities for the reptile species in this study are shown in Table 1. The full set of ranked candidate models from which these estimates were derived is presented in Tables S3, S4 & S5. Within each data set, 15 models were run for each species, except for slow-worms in the two 29-site data sets, given that the species was detected at all sites. The naïve occupancy, i.e. the proportion of sites where the species was detected at least once, is also shown for reference in Table 1. This quantity provides a lower bound as it assumes that the species has not been missed at any of the occupied sites. In this analysis the naïve occupancy was in all cases very close to the estimated occupancy suggesting that after the six survey rounds the species had been detected in most occupied sites, i.e. p* was close to unity. Estimated occupancy ranged from 1.00 (slow-worm in both 29 site data sets) down to 0.14 (sand lizard in the 45 site data set). Detection probability varied from a high of 0.91 (slow-worm, 2009 29 site data set), down to 0.25 (sand lizard, 2010, 29 sites data set). For no species was a site-specific variable found to be consistently significant as a determinant of occupancy.

**Table 1:** Naïve occupancy plus estimated occupancy (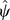) and detection probabilities (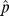) for each data set.

Species	Naïve 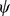	 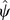 (s.e.)		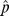 *(s.e.)*	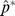
**2009, 29 sites**					
Slow-worm	1.00	1.00(−)		0.91(0.037)	1.00
Common lizard	0.79	0.80(0.076)		0.67(0.042)	1.00
Sand lizard	0.21	0.21(0.080)		0.42(0.108)	0.96
Adder	0.79	0.81(0.071)		0.55(0.045)	0.99
Grass snake	0.72	0.73(0.084)		0.53(0.058)	0.99
Smooth snake	0.21	0.21(0.076)		0.79(0.084)	1.00
**2010, 29 sites**					
Slow-worm	1.00	1.00 (−)		0.82(0.061)	1.00
Common lizard	0.79	0.81(0.078)		0.59(0.047)	0.99
Sand lizard	0.22	0.32(0.136)		0.25(0.098)	0.94
Adder	0.76	0.76(0.080)		0.56(0.049)	0.99
Grass snake	0.79	0.81(0.078)		0.59(0.049)	0.99
Smooth snake	0.24	0.24(0.080)		0.64(0.069)	1.00
**2010, 45 sites**					
Slow-worm	0.87	0.87(0.051)		0.82(0.053)	1.00
Common lizard	0.76	0.76(0.066)		0.63(0.037)	1.00
Sand lizard	0.13	0.14(0.048)		0.42(0.093)	0.93
Adder	0.56	0.56(0.075)		0.57(0.048)	0.99
Grass snake	0.64	0.67(0.075)		0.43(0.042)	0.97
Smooth snake	0.16	0.16(0.054)		0.64(0.067)	1.00

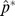
 is the probability of detecting a species at an occupied site on at least one of the six survey visits carried out.

Some of the models listed in Tables S3, S4 and S5 involved a survey-specific *p*, that is the estimated detection probability varied between the individual survey rounds (i.e. months). Where these models accounted for >0.50 of the AIC weight the monthly variation is listed in Table S6. Slow-worm in both 2010 data sets (Tables S4 and S5) is the only affected species. While the results did suggest a monthly variation, there was not a significant or consistent pattern except for low detection rates in March.

### Survey Design Recommendations

According to the estimated detection probabilities, the number of survey visits required to determine species presence at an occupied site with a given certainty was generally one or two for 80% confidence, two or three for 90% and three or four for 95% (Table 2). The main exception to this general rule was the sand lizard, with up to ten surveys required for 95% confidence. In particular, the low estimate of detection (Table 1), and consequent high estimate of the number of visits in the 2010 29 sites data set (Table 2) requires some examination. An examination of the entry for sand lizards in Table S4 reveals that a single model, *ψ* (.), *p*(experience), with high AIC weight dominated this particular analysis. This model suggests a larger proportion of occupied sites than other models in the same data set, and a lower detection rate. Without this model, the results are broadly similar to those in the other two data sets.

**Table 2:** Number of surveys required to determine species presence at occupied sites.

80% confidence level			
Species	2009, 29 sites	2010, 29 sites	2010, 45 sites
Slow-worm	1 (0.4–0.9)	1 (0.6–1.3)	1 (0.6–1.3)
Common lizard	1 (1.2–1.8)	2 (1.4–2.3)	2 (1.3–2.0)
Sand lizard	3 (1.6–6.9)	6 (2.8–27.0)	3 (1.7–5.9)
Adder	2 (1.6–2.6)	2 (1.5–2.6)	2 (1.5–2.5)
Grass snake	2 (1.6–3.0)	2 (1.4–2.4)	3 (2.2–3.8)
Smooth snake	1 (0.5–1.6)	2 (1.1–2.3)	1 (1.1–2.3)
**90% confidence level**			
**Species**	**2009, 29 sites**	**2010, 29 sites**	**2010, 45 sites**
Slow-worm	1 (0.6–1.3)	1 (0.8–1.9)	1 (0.9–1.8)
Common lizard	2 (1.6–2.6)	3 (2.0–3.3)	2 (1.9–2.8)
Sand lizard	4 (2.3–9.9)	8 (3.9–38.6)	5 (2.5–8.5)
Adder	3 (2.3–3.7)	3 (2.2–3.7)	3 (2.1–3.6)
Grass snake	3 (2.2–4.3)	3 (2.0–3.4)	5 (3.2–5.4)
Smooth snake	1 (0.7–2.3)	2 (1.5–3.3)	2 (1.7–2.9)
**95% confidence level**			
**Species**	**2009, 29 sites**	**2010, 29 sites**	**2010, 45 sites**
Slow-worm	1 (0.7–1.6)	2 (1.1–2.5)	2 (1.2–2.4)
Common lizard	3 (2.1–3.4)	3 (2.5–3.7)	3 (2.5–3.7)
Sand lizard	6 (3.0–12.8)	10 (5.1–50.2)	6 (3.3–11.0)
Adder	4 (2.9–4.8)	4 (2.8–4.8)	4 (2.7–4.6)
Grass snake	4 (2.9–5.6)	3 (2.6–4.4)	5 (4.2–7.0)
Smooth snake	2 (1.–3.1)	3 (2.0–4.3)	3 (2.0–4.2)

Data provided at three levels of certainty, with 95% confidence intervals in parentheses.

**Figure 1:**
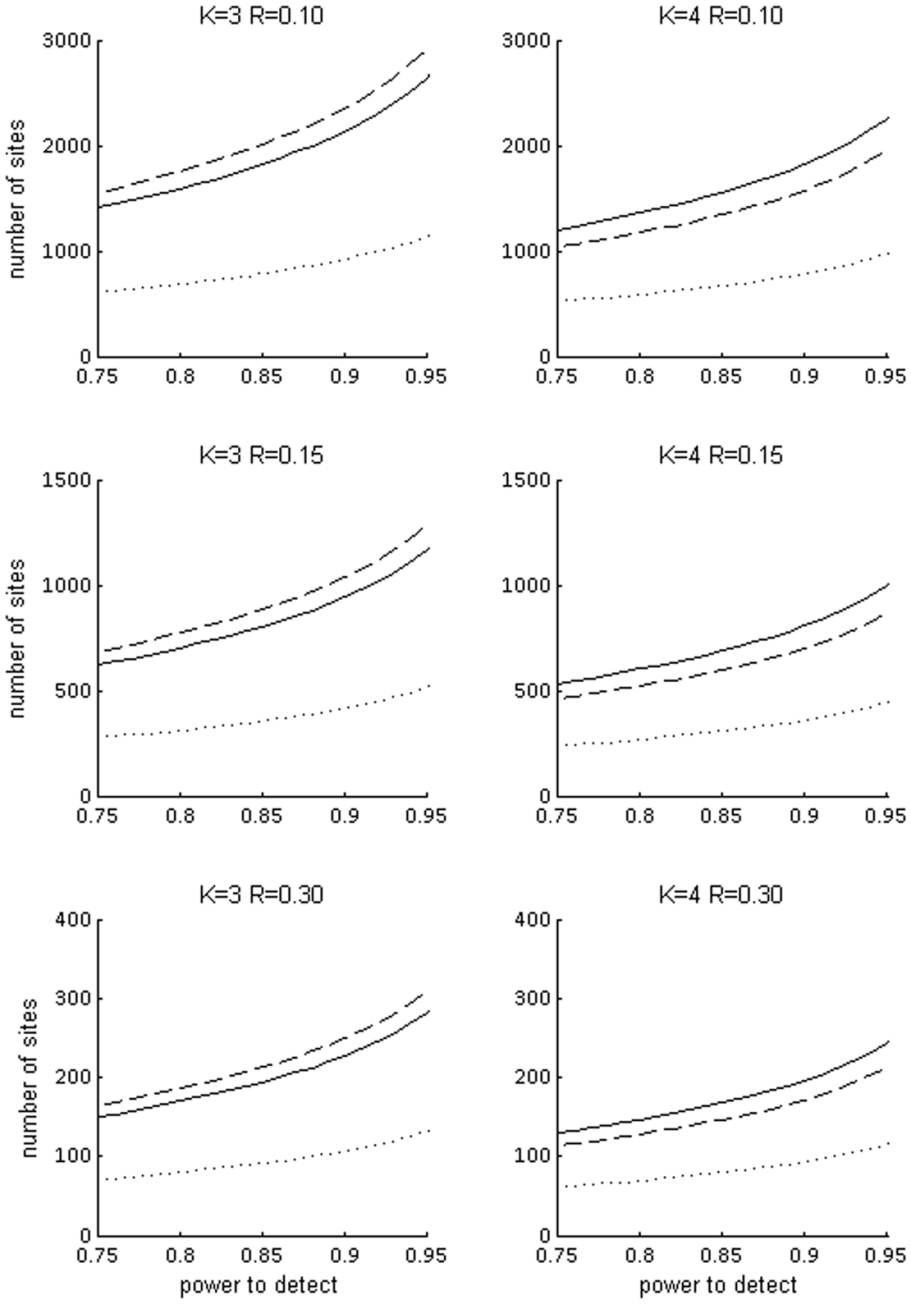
Number of sites to detect an occupancy decline with a given power.

Based on the occupancy and detection probabilities estimated for the 2010 45-site data set, the optimal number of survey visits to minimize the variance of the occupancy estimator was two for slow-worms, three for adders and common lizards and four for grass snakes. The number of sites that would be required in a subsequent survey programme to detect an occupancy decline with a given power varied considerably among species (Figure S2). Being a very common, easy-to-detect, species (high *ψ* and *p*) the slow-worm requires substantially less effort and any design that fulfils the requirements for the other species should be able to detect a decline in slow-worm occupancy. Figure 1 represents the number of sites to survey for the three remaining widespread species assuming three or four survey visits per site, i.e. the two optimal replication values obtained for species within this group. At *K* = 3 visits, the design requirements derived for grass snake are the more restrictive, that is, this species requires the largest number of sites to achieve the target power to detect a decline, compared with the other two. At *K* = 4 visits, the adder is the more demanding species. The total effort required taking into account all the species is very similar regardless of whether the number of replicates is 3 or 4 (Figure S3), driven by grass snake or adder respectively. The results indicate that, for instance, in order to have a 85% power to detect a decline (at α  = 0.05) in any of the four more common species when the true decline is 15%, a suitable design would require carrying out 3 repeated surveys at a total of 886 sampling sites, or 4 repeated surveys at a total of 688 sites. The number of sampling sites reduces to 212 and 167 respectively if the target is to detect a true decline of 30%. The simulations showed results consistent with those derived from eq 3 (Table S7). Of course the number of sites decreases if the significance level α (i.e. probability of type I error) is relaxed.

## Discussion

### Comparison with Previous Studies

Previous studies have investigated the detectability of some of the species examined here. For grass snakes, detection probabilities of between 0.11–0.25 were determined [22] compared with our 0.44–0.58 in Table 1. For slow-worms an estimated detection probability of approximately 0.34 [33] compared to our 0.83–0.91 in Table 1. Species detectability is the result of many factors and comparisons across studies have to be made with care as, for instance, in different geographical areas habitat characteristics or population abundance may differ. It is nevertheless interesting to note that a key difference between our study and these earlier ones was our use of ACOs, in addition to directed transects, to detect reptiles. The other studies relied on visual transects only, and yielded noticeably lower detection probabilities.

The use of ACOs in our study yielded a considerable number of detections for most species and was particularly effective for slow-worms, smooth snakes and grass snakes (Table S2). An earlier study [34] also found increases in recording rates by the use of ACOs, and that sand lizards were less likely to use ACOs than other species. Use of ACOs has the added advantage that animals are often seen at very close quarters, facilitating correct identification, which may be an issue amongst less experienced surveyors [35]. Distinction between detectability and availability may be made, on the basis that some individuals may not be available for detection at any given time, including any outside the area surveyed, or underground [32]. ACOs effectively increase the availability, and therefore detectability, of those individuals that behave in such a manner. We therefore recommend the use of ACOs both to increase the detection rate of most reptiles in temperate climates and also to reduce misidentification.

### Site-specific Variables

The lack of consistent, significant site-specific variables as determinants of occupancy in our models may suggest that inappropriate variables were recorded, that complex mixes of site variables determine occupancy, or simply that the sample size was too small for the potential effect size to be detected. It is also possible that in the highly fragmented landscapes where much of the fieldwork occurred, the likelihood of occupation was affected by random factors. Small sites usually support small populations and, if local extinction occurs, natural re-colonization may be prevented by barriers of unsuitable habitat.

Some species in this study were habitat generalists. Slow-worms were found in all sites within the 29- site data sets, and most sites in the 45- sites data set. Therefore their distribution did not depend on site characteristics. Conversely, sand lizards and smooth snakes have very limited habitat preferences in the UK, and their specialized requirements may not be fully reflected by the covariates used in this study. Both species are strongly associated with mature dry heathland [36], [37], a specialized habitat type not examined individually within this study.

### Detection Probability and Survey Effort to Ensure Detection

Detection probability for slow-worms showed variability through the survey season (Table S6), and was notably low in March. In the UK, this is when reptiles emerge from hibernation. Some of the models in Tables S3, S4 and S5 also showed temperature affecting the estimate of *p*. These were probably capturing the same effect, as temperature varied through the survey season. Certainly temperature has been shown to affect detectability of reptiles in similar studies [35]. It is therefore advisable to delay surveys until April, when detectability increases. However, work could begin in March if suitable (i.e. warm) weather conditions prevail. Spreading surveys across the whole season should limit the effects of such variations.

Survey duration was not a covariate consistently affecting detection for most species. The exception to this general rule was common lizard, where survey duration was a factor affecting detectability in all three data sets (Tables S3, S4 and S5). Increasing the duration of a survey may increase the availability for detection for this species as surveyors search a longer transect [33]. Increasing the number of ACOs on a site is likely to have a similar effect. However, the cost of increasing the duration of each survey is likely to be that fewer surveys are possible within a given time frame. Given the relatively high detection rate of this species in Tables S3, S4, S5 we do not consider such a trade-off is worthwhile.

For most species three to four surveys were sufficient for 95% confidence that if a species is present, it will be located. However, it should be noted that the detection probabilities given carry uncertainty, and allowance for this should be incorporated into the survey design. Most species will vary in detectability between individual sites, with small populations requiring more effort to detect than larger populations [38].

### Design for Future Surveys

Our survey design focused primarily on the four reptile species with a wide distribution within the UK. Our sample size recommendations for trend detection are based on the recognition that the number of sites sampled will be much smaller than the total number of sites available within the distribution range of these species, also known as the superpopulation [39]. The overall objective of the monitoring is to estimate any changes in the probability of occupancy for the species across their range (i.e. in the superpopulation) and therefore the study design needs to account for the stochasticity introduced by sampling from a larger population. Due to their limited distribution within the UK, sand lizards and smooth snakes can be treated differently, with more comprehensive monitoring than species having a wide national range. A specific monitoring system for these two species has, in fact, been in existence for over 20 years and is coordinated in the UK by ARC (Amphibian and Reptile Conservation), (http://www.narrs.org.uk/monitoringss.html and http://www.narrs.org.uk/sandlizard_monitoring.htm, both accessed February 14th 2012). The recommendations on number of survey visits to ensure detection with a given probability determined in the present study provide a useful indication as to how intensively sites with sand lizards and smooth snakes should be monitored.

The total effort required according to the results of the power analysis was very similar regardless of whether three or four surveys per site were carried out. Which design is chosen may depend on a number of factors, such as the distribution and availability of surveyors, or the particular species of interest. In the UK there is currently concern about the apparent decline of the adder (http://www.adder.org.uk/accessed February 14th 2012). Therefore the three repeat survey protocol may be the better one to adopt as it gives the most precise results for a given total effort for the species currently of greatest interest.

The derived design recommendations are based on the current estimates of occupancy and detectability, and should therefore be re-evaluated as these change. It is also important to bear in mind that the system will often be more complex than described by the model, which implies that the true power of a design may differ from its theoretical power. In our study we have considered occupancy as a state-variable and have used the basic occupancy model [19] for analysis. Abundance-induced heterogeneity in detection probability can induce bias in the occupancy estimator [40]. If such heterogeneity is suspected, a model that accounts for it would be more appropriate [41]. In fact, since count data can be collected in these surveys, rather than restricting the analysis to occupancy estimation from detection/non-detection data, abundance could be estimated from the replicated counts [42].

With the biodiversity crisis likely to deepen, the effective deployment of available expertise and resources will become increasingly important. In the developing world long term biodiversity monitoring schemes may only be sustainable if volunteers are used [43]. Indeed, such schemes are increasing in number, especially in Asia [44], [45]. Despite concerns over the reliability of data collected by volunteers [46], [47], [48], volunteer networks provide a workforce that can collect essential biodiversity data that would otherwise be beyond the reach of professional scientists or conservation practitioners. However, this is subject to the provisos that adequate training is given in the survey methods and that sufficient repeat surveys using suitable methods are carried out at a sufficiently large number of sites. Here we have shown how ‘sufficient repeat surveys’ and ‘sufficiently large number of sites’ may be quantified. For presence-absence surveys of species that may be relatively easy to identify when detected, the design procedure described here allows optimizing surveys so that population declines can be identified more effectively.

## Supporting Information

Figure S1 **Sites location map.**
(TIF)

Figure S2 **Number of sites required to detect a decline.**
(TIF)

Figure S3 **Total effort required to detect a decline.**
(TIF)

Table S1 Number of sites where species were encountered by month, year and sample size.
(DOC)

Table S2 Number of encounters by species, dataset and method of detection.
(DOCX)

Table S3 Occupancy models from the 2009 29 sites dataset.
(PDF)

Table S4 Occupancy models from the 2010 29 sites dataset.
(PDF)

Table S5 Occupancy models from the 2010 45 sites dataset.
(PDF)

Table S6 Monthly variation in detection probability.
(PDF)

Table S7 Power simulation results.
(DOCX)
