# Supplementary figures and images for: When Is a Species Declining? Optimizing Survey Effort to Detect Population Changes in Reptiles
Source: PLoS One. 2012 Aug 22;7(8):e43387. doi: 10.1371/journal.pone.0043387 (PMC3425567; doi:10.1371/journal.pone.0043387)

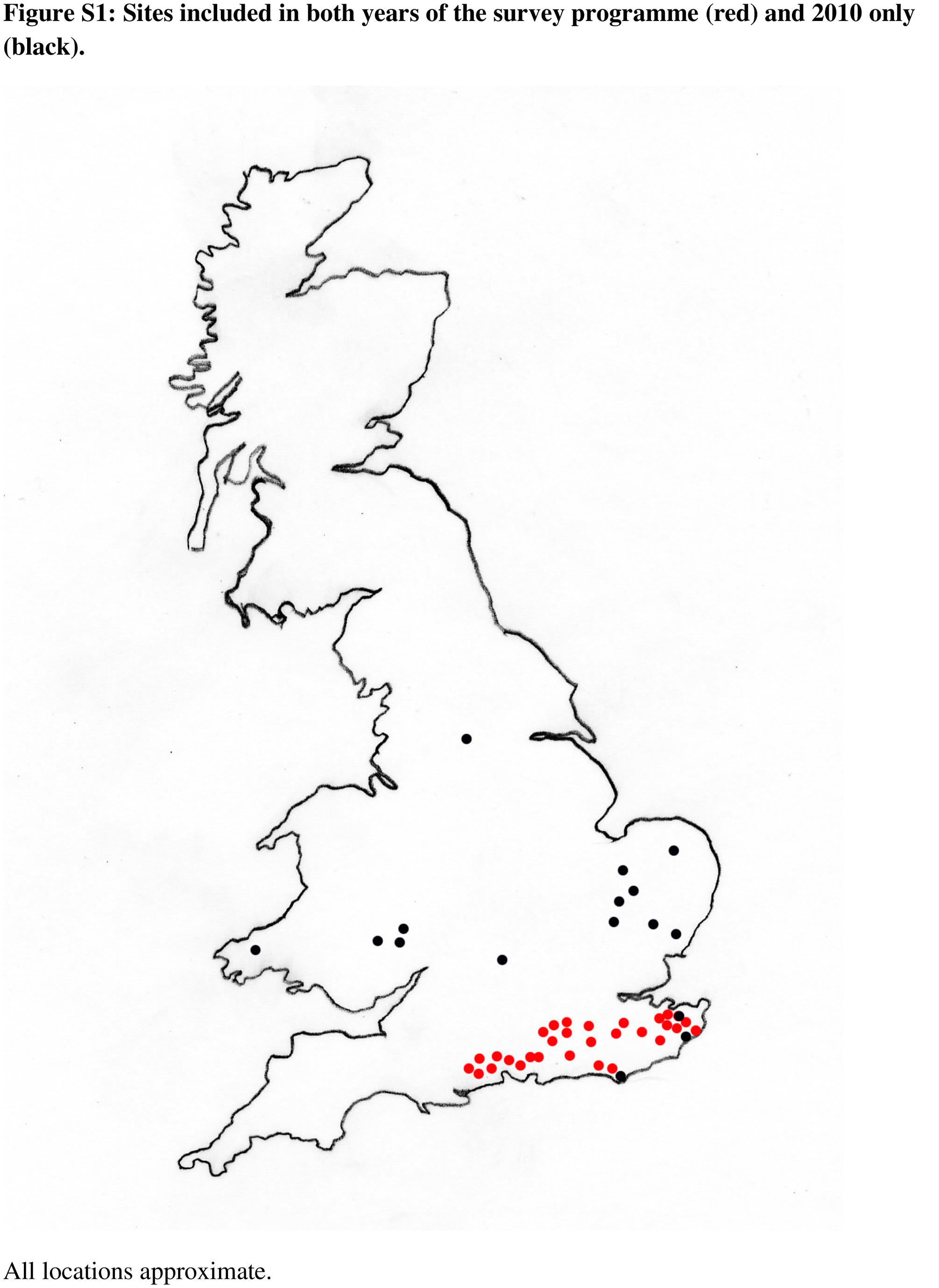

Supplement: Figure S1 — Sites location map. (TIF) [file pone.0043387.s001.tif]

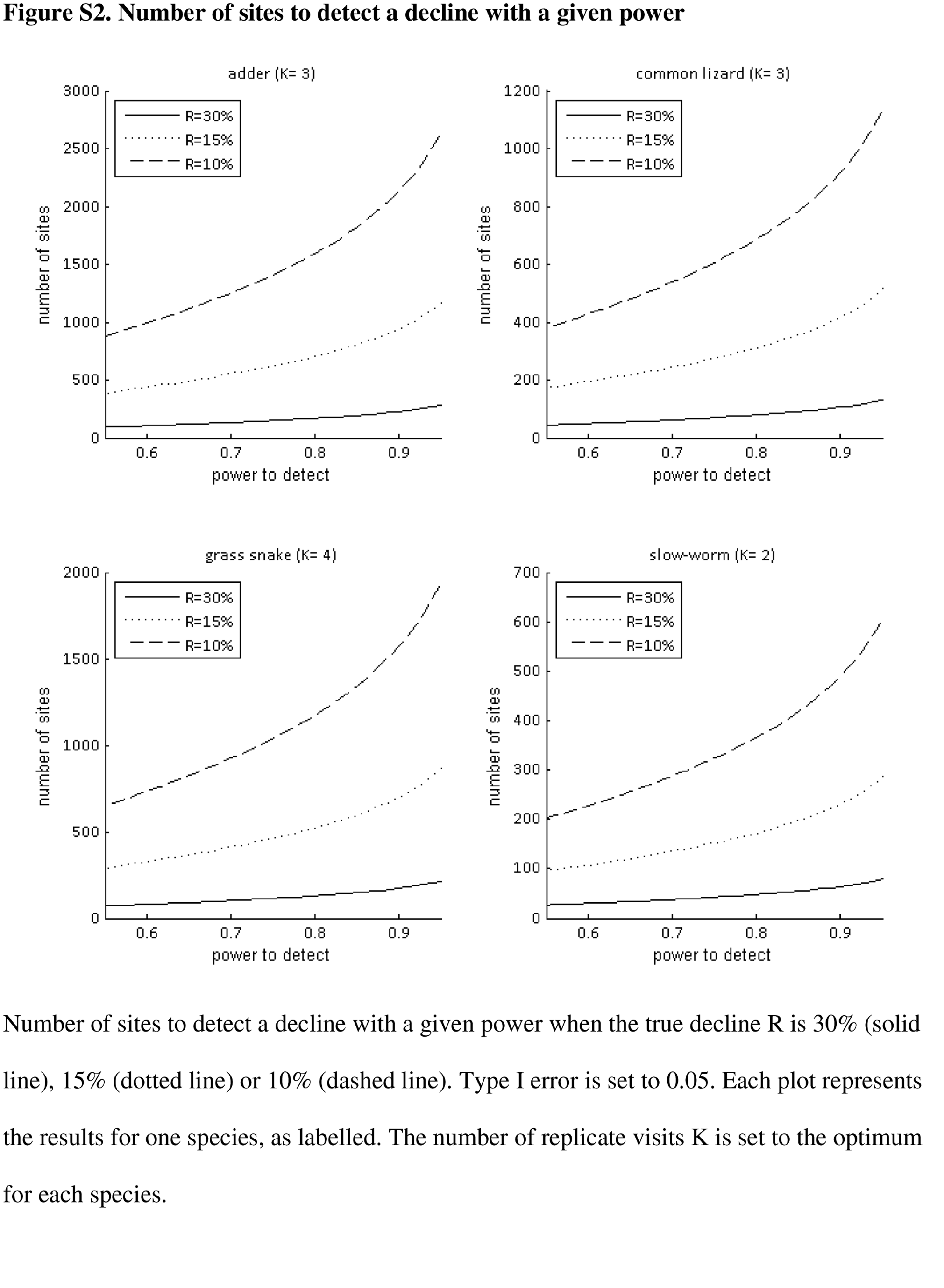

Supplement: Figure S2 — Number of sites required to detect a decline. (TIF) [file pone.0043387.s002.tif]

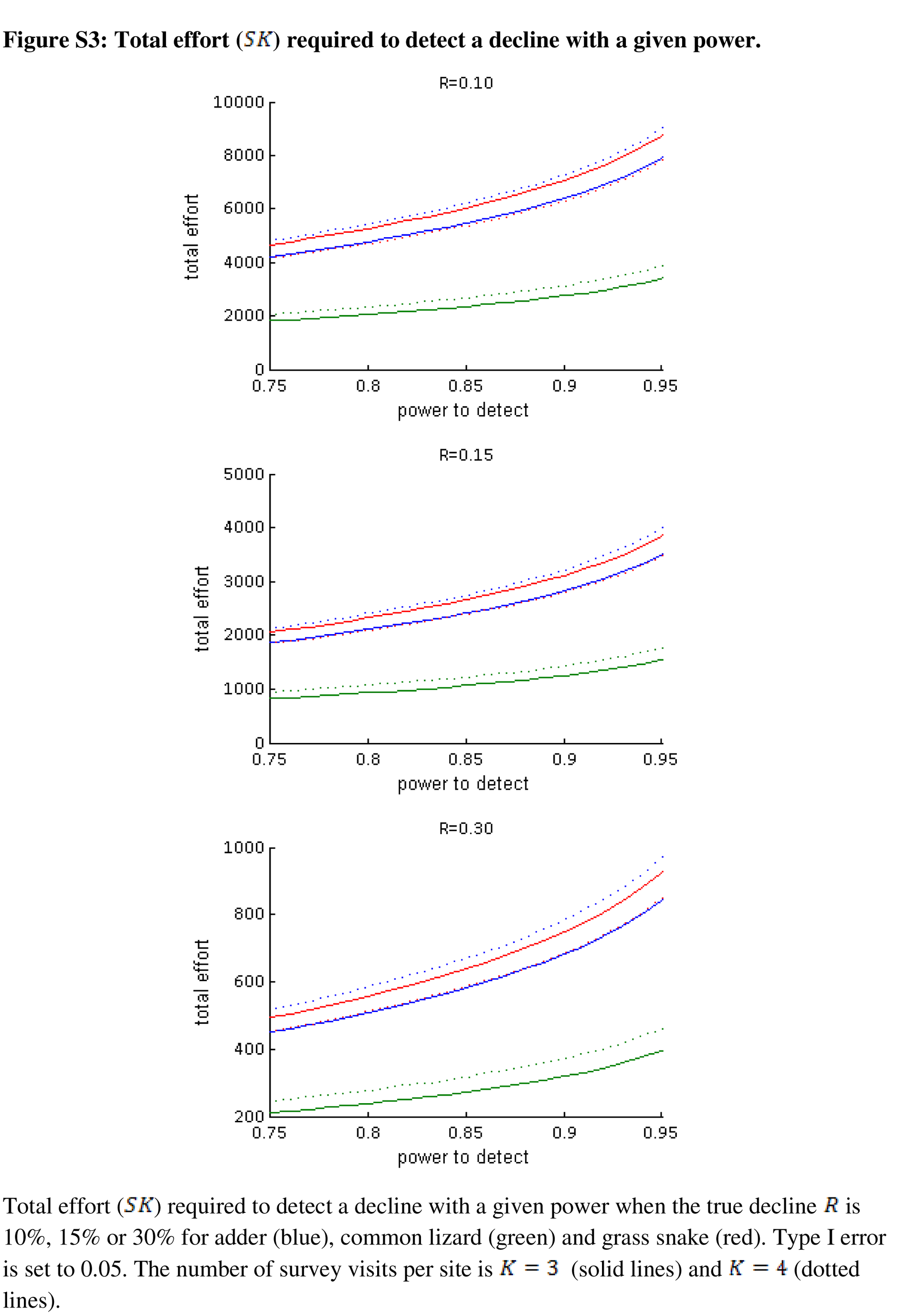

Supplement: Figure S3 — Total effort required to detect a decline. (TIF) [file pone.0043387.s003.tif]
