# Supplementary material for: When Is a Species Declining? Optimizing Survey Effort to Detect Population Changes in Reptiles
Source: PLoS One. 2012 Aug 22;7(8):e43387. doi: 10.1371/journal.pone.0043387 (PMC3425567; doi:10.1371/journal.pone.0043387)
Supplement: Table S1 — Number of sites where species were encountered by month, year and sample size. (DOC) [file pone.0043387.s004.doc]

**Table S1: Number of sites where species were encountered by month, year and sample size.**

|  |  |  | 2009, 29 sites | |  |  |  |
| --- | --- | --- | --- | --- | --- | --- | --- |
|  |  |  |  |  |  |  |  |
| Species | Mar | Apr | May | Jun | Jul | Sep | Total |
| Slow-worm | 21 | 26 | 29 | 28 | 26 | 26 | 29 |
| Common lizard | 15 | 16 | 19 | 14 | 12 | 16 | 23 |
| Sand lizard | 2 | 4 | 4 | 2 | 0 | 3 | 6 |
| Adder | 13 | 9 | 12 | 13 | 16 | 12 | 23 |
| Grass snake | 7 | 11 | 13 | 11 | 17 | 8 | 21 |
| Smooth snake | 2 | 4 | 6 | 5 | 6 | 4 | 6 |
|  |  |  |  |  |  |  |  |
|  |  |  | 2010, 29 sites | |  |  |  |
|  |  |  |  |  |  |  |  |
| Species | Mar | Apr | May | Jun | Jul | Sep | Total |
| Slow-worm | 12 | 28 | 26 | 24 | 23 | 28 | 29 |
| Common lizard | 14 | 18 | 14 | 12 | 9 | 16 | 23 |
| Sand lizard | 1 | 3 | 3 | 3 | 3 | 3 | 6 |
| Adder | 10 | 17 | 14 | 13 | 11 | 8 | 22 |
| Grass snake | 2 | 9 | 9 | 12 | 11 | 13 | 19 |
| Smooth snake | 0 | 4 | 5 | 6 | 5 | 6 | 7 |
|  |  |  |  |  |  |  |  |
|  |  |  | 2010, 45 sites | |  |  |  |
|  |  |  |  |  |  |  |  |
| Species | Mar | Apr | May | Jun | Jul | Sep | Total |
| Slow-worm | 18 | 36 | 35 | 32 | 32 | 38 | 39 |
| Common lizard | 21 | 27 | 23 | 19 | 14 | 23 | 34 |
| Sand lizard | 1 | 3 | 3 | 3 | 3 | 3 | 6 |
| Adder | 11 | 20 | 15 | 15 | 14 | 10 | 25 |
| Grass snake | 3 | 13 | 14 | 18 | 15 | 16 | 29 |
| Smooth snake | 0 | 4 | 5 | 6 | 5 | 6 | 7 |

The Total column sums the number of sites where each species was detected at least once.
