# Supplementary material for: When Is a Species Declining? Optimizing Survey Effort to Detect Population Changes in Reptiles
Source: PLoS One. 2012 Aug 22;7(8):e43387. doi: 10.1371/journal.pone.0043387 (PMC3425567; doi:10.1371/journal.pone.0043387)
Supplement: Table S2 — Number of encounters by species, dataset and method of detection. (DOCX) [file pone.0043387.s005.docx]

**Table S2: Number of encounters by species, data set and method of detection (i.e. transect and/or artificial cover objects).**

| 29 sites, 2009 | | | |
| --- | --- | --- | --- |
|  | Transects | Refugia | Total |
| Slow-worm | 8 | 156 | 156 |
| Common lizard | 66 | 58 | 92 |
| Sand lizard | 14 | 1 | 15 |
| Adder | 52 | 36 | 75 |
| Grass snake | 14 | 58 | 67 |
| Smooth snake | 3 | 27 | 27 |
| 29 sites, 2010 | | | |
|  | Transects | Refugia | Total |
| Slow-worm | 13 | 141 | 141 |
| Common lizard | 51 | 81 | 83 |
| Sand lizard | 15 | 4 | 16 |
| Adder | 41 | 46 | 73 |
| Grass snake | 8 | 51 | 56 |
| Smooth snake | 3 | 26 | 26 |
| 45 sites, 2010 | | | |
|  | Transects | Refugia | Total |
| Slow-worm | 20 | 190 | 191 |
| Common lizard | 78 | 126 | 127 |
| Sand lizard | 15 | 4 | 16 |
| Adder | 49 | 55 | 84 |
| Grass snake | 12 | 74 | 79 |
| Smooth snake | 3 | 26 | 26 |

The term ‘encounter’ denotes the number of survey occasions on which the species was positively identified, not the number of individuals of each species. Sites in each data set were surveyed 6 times. For reasons of space, artificial cover objects are referred to by the alternative name of refugia within the Table.
