# Supplementary material for: When Is a Species Declining? Optimizing Survey Effort to Detect Population Changes in Reptiles
Source: PLoS One. 2012 Aug 22;7(8):e43387. doi: 10.1371/journal.pone.0043387 (PMC3425567; doi:10.1371/journal.pone.0043387)
Supplement: Table S4 — Occupancy models from the 2010 29 sites dataset. (PDF) [file pone.0043387.s007.pdf]

Table S4: Occupancy models by species from the 2010, 29 sites data set.

Species & Model	N	-2 log likelihood	ΔAIC	AIC Weight	$\hat{\psi}(s.e)$	$\hat{p}(s.e)$
Slow-worm						
ψ(.), p(survey-specific)	7	130.02	0.00	1.00	1.00(-)*	0.82 (0.061)
ψ(.), p(.)	2	162.28	22.26	0.00	1.00(-)*	0.82 (0.030)
ψ(.), p(air temp)	3	161.81	23.79	0.00	1.00(-)*	0.82 (0.030)
				Model averaged:	1.00(-)*	0.82 (0.061)
Common lizard						
ψ(.), p(duration)	3	206.45	0.00	0.56	0.81 (0.077)	0.58 (0.044)
ψ(.), p(soil temp)	3	210.35	3.90	0.08	0.80 (0.076)	0.60 (0.042)
ψ(area), p(.)	3	210.59	4.14	0.07	0.80 (0.073)	0.60 (0.042)
ψ(.), p(.)	2	213.09	4.64	0.05	0.80 (0.076)	0.60 (0.043)
ψ(.), p(survey-specific)	7	204.02	5.57	0.03	0.80 (0.076)	0.60 (0.099)
ψ(.), p(air temp)	3	212.06	5.61	0.03	0.80 (0.076)	0.61 (0.042)
ψ(.), p(cloud cover)	3	212.44	5.99	0.03	0.80 (0.076)	0.60 (0.043)
ψ(.), p(refugia)	3	212.54	6.09	0.03	0.80 (0.076)	0.60 (0.043)
ψ(level), p(.)[a]	3	212.82	6.37	0.02	0.80 (0.075)	0.60 (0.043)
ψ(level & south facing), p(.)[a]	3	212.84	6.39	0.02	0.80 (0.075)	0.60 (0.043)
ψ(soil type), p(.)	5	208.89	6.44	0.02	0.80 (0.117)	0.60 (0.043)
ψ(.), p(experience)	5	208.95	6.50	0.02	0.80 (0.076)	0.59 (0.067)
ψ(south facing), p(.)[a]	3	213.06	6.61	0.02	0.80 (0.076)	0.60 (0.043)
ψ(connectivity), p(.)	6	211.14	10.69	0.00	0.80 (0.144)	0.60 (0.043)
ψ(human impact), p(.)	7	211.52	13.07	0.00	0.80 (0.145)	0.60 (0.043)
				Model averaged:	0.81 (0.078)	0.59 (0.047)

Species & Model	N	-2 log likelihood	ΔAIC	AIC Weight	$\hat{\psi}(s.e)$	$\hat{p}(s.e)$
Sand lizard						
ψ(.), p(experience)	5	62.25	0.00	0.94	0.33 (0.136)	0.24 (0.093)
ψ(.), p(air temp)	3	73.35	7.10	0.03	0.22 (0.083)	0.37 (0.090)
ψ(.), p(soil temp)	3	76.08	9.83	0.01	0.21 (0.078)	0.43 (0.085)
ψ(.), p(.)	2	78.61	10.36	0.01	0.21 (0.079)	0.43 (0.079)
ψ(.), p(duration)	3	77.01	10.76	0.00	0.26 (0.119)	0.43 (0.085)
ψ(.), p(cloud cover)	3	78.07	11.82	0.00	0.21 (0.078)	0.44 (0.089)
ψ(south facing), p(.)[a]	3	78.47	12.22	0.00	0.21 (0.078)	0.43 (0.089)
ψ(area), p(.)[a]	3	78.52	12.27	0.00	0.21 (0.078)	0.43 (0.089)
ψ(level & south facing), (.)[a]	3	78.54	12.29	0.00	0.21 (0.078)	0.43 (0.089)
ψ(level), p(.)[a]	3	78.60	12.35	0.00	0.21 (0.078)	0.43 (0.089)
ψ(.), p(refugia)[a]	3	78.61	12.36	0.00	0.21 (0.079)	0.43 (0.091)
ψ(connectivity), p(.)	6	74.97	14.72	0.00	0.21 (0.141)	0.43 (0.089)
ψ(.), p(Survey-specific)	7	76.22	17.97	0.00	0.21 (0.078)	0.43 (0.195)
				Model averaged:	0.32 (0.136)	0.25 (0.098)
Adder						
ψ(level & south facing), p(.)	3	206.71	0.00	0.22	0.76 (0.076)	0.56 (0.045)
ψ(.), p(soil temp)	3	207.96	1.25	0.12	0.76 (0.080)	0.56 (0.044)
ψ(.), p(.)	2	209.98	1.27	0.12	0.76 (0.080)	0.56 (0.045)
ψ(south facing), p(.)	3	208.40	1.69	0.09	0.76 (0.078)	0.56 (0.045)
ψ(.), p(air temp)	3	208.58	1.87	0.09	0.76 (0.080)	0.56 (0.044)
ψ(.), p(duration)	3	208.84	2.13	0.08	0.76 (0.081)	0.55 (0.046)
ψ(.), p(survey-specific)	7	201.07	2.36	0.07	0.76 (0.080)	0.56 (0.103)
ψ(.), p(cloud cover)	3	209.15	2.44	0.06	0.76 (0.080)	0.56 (0.045)
ψ(.), p(refugia)	3	209.50	2.79	0.05	0.77 (0.080)	0.55 (0.045)
ψ(area), p(.)[a]	3	209.87	3.16	0.05	0.76 (0.080)	0.56 (0.045)
ψ(level), p(.)[a]	3	209.94	3.23	0.04	0.76 (0.080)	0.56 (0.045)
ψ(.), p(experience)	5	209.31	6.60	0.01	0.76 (0.080)	0.57 (0.071)
ψ(soil type), p(.)	5	209.34	6.63	0.01	0.76 (0.137)	0.56 (0.045)
ψ(connectivity), p(.)	6	208.82	8.11	0.00	0.76 (0.155)	0.56 (0.045)
				Model averaged:	0.76 (0.080)	0.56 (0.049)

Species & Model	N	-2 log likelihood	ΔAIC	AIC Weight	$\hat{\psi}(s.e)$	$\hat{p}(s.e)$
Grass snake						
ψ(.), p(duration)	3	206.45	0.00	0.56	0.81 (0.077)	0.58 (0.044)
ψ(.), p(soil temp)	3	210.35	3.90	0.08	0.80 (0.076)	0.60 (0.042)
ψ(area), p(.)	3	210.59	4.14	0.07	0.80 (0.073)	0.60 (0.042)
ψ(.), p(.)	2	213.09	4.64	0.05	0.80 (0.076)	0.60 (0.043)
ψ(.), p(survey-specific)	7	204.02	5.57	0.03	0.80 (0.076)	0.60 (0.099)
ψ(.), p(air temp)	3	212.06	5.61	0.03	0.80 (0.076)	0.61 (0.042)
ψ(.), p(cloud cover)	3	212.44	5.99	0.03	0.80 (0.076)	0.60 (0.043)
ψ(.), p(refugia)	3	212.54	6.09	0.03	0.80 (0.076)	0.60 (0.043)
ψ(.), p(experience)	5	208.68	6.23	0.02	0.80 (0.076)	0.59 (0.081)
ψ(level), p(.)[a]	3	212.82	6.37	0.02	0.80 (0.075)	0.60 (0.075)
ψ(level & south facing), p(.)[a]	3	212.84	6.39	0.02	0.80 (0.075)	0.60 (0.075)
ψ(soil), p(.)	5	208.89	6.44	0.02	0.80 (0.117)	0.60 (0.043)
ψ(south facing), p(.)[a]	3	213.06	6.61	0.02	0.80 (0.076)	0.60 (0.043)
ψ(connectivity), p(.)	6	211.14	10.69	0.00	0.80 (0.144)	0.60 (0.043)
				Model averaged:	0.81 (0.078)	0.59 (0.049)
Smooth snake						
ψ(.), p(soil temp)	3	73.43	0.00	0.89	0.24 (0.080)	0.64 (0.062)
ψ(.), p(survey-specific)	7	69.84	4.41	0.10	0.24 (0.080)	0.62 (0.132)
ψ(.), p(refugia)	3	84.10	10.67	0.00	0.24 (0.080)	0.67 (0.070)
ψ(area), p(.)	3	85.33	11.90	0.00	0.24 (0.076)	0.62 (0.076)
ψ(.), p(.)	2	87.82	12.39	0.00	0.24 (0.080)	0.62 (0.076)
ψ(level & south facing), p(.)	3	86.16	12.73	0.00	0.24 (0.077)	0.62 (0.076)
ψ(.), p(air temp)	3	86.63	13.20	0.00	0.24 (0.080)	0.59 (0.081)
ψ(south facing), p(.)	3	86.82	13.39	0.00	0.24 (0.078)	0.62 (0.076)
ψ(.), p(duration)	3	86.98	13.55	0.00	0.24 (0.080)	0.67 (0.089)
ψ(.), p(cloud cover)[a]	3	87.75	14.32	0.00	0.24 (0.080)	0.62 (0.076)
ψ(level), p(.)[a]	3	87.78	14.35	0.00	0.24 (0.080)	0.62 (0.076)
ψ(connectivity), p(.)	6	84.37	16.94	0.00	0.24 (0.144)	0.62 (0.076)
				Model averaged:	0.24 (0.080)	0.64 (0.069)

N = Number of parameters in the model, $\hat{\psi}$ = estimated occupancy probability, and $\hat{p}$ = estimated detection probability. Models that failed to converge were discarded. 15 models were therefore run for each species in this data set, except for slow-worm where occupancy was known to be 1.00 and models with occupancy covariates were not therefore run, reducing the number of candidate models for this species to 8. Model notation used is:

(.)	The parameter was a constant	(air temp)	Mean air temperature during surveys
(survey-specific)	The parameter varied over time	(soil temp)	Mean soil temperature during surveys
(area)	The area of the site measured in hectares	(cloud cover)	Proportion of the sky covered by cloud
(connectivity)	The connectivity of the site with other areas of habitat	(duration)	Length of the survey in minutes
(human impact)	The amount of human activity at the site	(refugia)	The total number of refugia checked
(level)	The proportion of the site that was level	(experience)	The field experience of each surveyor
(south facing)	The proportion of the site that faced south		
(level & south facing)	The proportions of the previous two categories combined		
(soil type)	Classification of site soil types into acid, alkaline or neutral		

Further details of covariates are given in the text.

* standard errors not computed as estimate is on the boundary of the parameter space; uncertainty is in any case small as the species was detected in all 29 sites.

[a] note that in these models the covariate can be considered to be acting as a "pretending variable" [30]
