# Supplementary material for: When Is a Species Declining? Optimizing Survey Effort to Detect Population Changes in Reptiles
Source: PLoS One. 2012 Aug 22;7(8):e43387. doi: 10.1371/journal.pone.0043387 (PMC3425567; doi:10.1371/journal.pone.0043387)
Supplement: Table S5 — Occupancy models from the 2010 45 sites dataset. (PDF) [file pone.0043387.s008.pdf]

**Table S5: Occupancy models by species from the 2010, 45 sites data set.**

| Model | $N$ | -2 log likelihood | Δ AIC | AIC Weight | $\hat{\psi}(s.e)$ | $\hat{p}(s.e)$ |
|---|---|---|---|---|---|---|
| Slow-worm | | | | | | |
| $\psi$(.), $p$(survey-specific) | 7 | 211.45 | 0.00 | 1.00 | 0.87 (0.051) | 0.82 (0.053) |
| $\psi$(.), $p$(soil temp) | 3 | 238.11 | 18.66 | 0.00 | 0.87 (0.051) | 0.82 (0.024) |
| $\psi$(.), $p$(refugia) | 3 | 238.34 | 18.89 | 0.00 | 0.87 (0.051) | 0.83 (0.025) |
| $\psi$(.), $p$(experience) | 6 | 237.95 | 24.50 | 0.00 | 0.87 (0.051) | 0.83 (0.041) |
| $\psi$(.), $p$(.) | 2 | 248.22 | 26.77 | 0.00 | 0.87 (0.051) | 0.83 (0.025) |
| $\psi$(area), $p$(.) | 3 | 246.70 | 27.25 | 0.00 | 0.87 (0.050) | 0.83 (0.025) |
| $\psi$(south facing), (.) | 3 | 247.46 | 28.01 | 0.00 | 0.87 (0.049) | 0.83 (0.025) |
| $\psi$(level), (.) | 3 | 247.60 | 28.15 | 0.00 | 0.87 (0.050) | 0.83 (0.025) |
| $\psi$(.), $p$(cloud cover) | 3 | 247.78 | 28.33 | 0.00 | 0.87 (0.051) | 0.83 (0.025) |
| $\psi$(.), $p$(air temp)[a] | 3 | 248.08 | 28.63 | 0.00 | 0.87 (0.051) | 0.83 (0.025) |
| $\psi$(.), $p$(duration)[a] | 3 | 248.19 | 28.74 | 0.00 | 0.87 (0.051) | 0.83 (0.025) |
| $\psi$ (level & south facing), (.)[a] | 3 | 248.21 | 28.76 | 0.00 | 0.87 (0.051) | 0.83 (0.025) |
| | | | | *Model averaged:* | 0.87 (0.051) | 0.82 (0.053) |
| Common lizard | | | | | | |
| $\psi$(.), $p$(duration) | 3 | 298.90 | 0.00 | 0.80 | 0.76 (0.065) | 0.63 (0.034) |
| $\psi$(.), $p$(survey-specific) | 7 | 296.16 | 5.26 | 0.06 | 0.76 (0.064) | 0.64 (0.081) |
| $\psi$ (soil type), (.) | 5 | 300.69 | 5.79 | 0.04 | 0.76 (0.097) | 0.64 (0.035) |
| $\psi$(.), $p$(.) | 2 | 308.22 | 7.32 | 0.02 | 0.76 (0.064) | 0.64 (0.035) |
| $\psi$(.), $p$(refugia) | 3 | 307.02 | 8.12 | 0.01 | 0.76 (0.064) | 0.64 (0.035) |
| $\psi$(area), $p$(.) | 3 | 307.24 | 8.34 | 0.01 | 0.76 (0.064) | 0.64 (0.035) |
| $\psi$(.), $p$(air temp) | 3 | 307.29 | 8.39 | 0.01 | 0.76 (0.064) | 0.64 (0.035) |
| $\psi$(.), $p$(soil temp) | 3 | 307.69 | 8.79 | 0.01 | 0.76 (0.064) | 0.64 (0.035) |
| $\psi$(.), $p$(cloud cover) | 3 | 307.71 | 8.81 | 0.01 | 0.76 (0.064) | 0.64 (0.035) |
| $\psi$(level), $p$(.)[a] | 3 | 307.78 | 8.88 | 0.01 | 0.76 (0.064) | 0.64 (0.035) |
| $\psi$(south facing), $p$(.)[a] | 3 | 308.08 | 9.18 | 0.01 | 0.76 (0.064) | 0.64 (0.035) |
| | | | | *Model averaged:* | 0.76 (0.066) | 0.63 (0.037) |

| Model | $N$ | -2 log likelihood | Δ AIC | AIC Weight | $\hat{\psi}(s.e)$ | $\hat{p}(s.e)$ |
|---|---|---|---|---|---|---|
| Sand lizard | | | | | | |
| $\psi$(soil type), $p$(.) | 5 | 70.06 | 0.00 | 0.85 | 0.14 (0.046) | 0.43 (0.089) |
| $\psi$(.), $p$(air temp) | 3 | 78.94 | 4.88 | 0.07 | 0.15 (0.057) | 0.35 (0.089) |
| $\psi$(.), $p$(soil temp) | 3 | 81.84 | 7.78 | 0.02 | 0.14 (0.053) | 0.44 (0.086) |
| $\psi$(.), $p$(.) | 2 | 84.32 | 8.26 | 0.01 | 0.14 (0.053) | 0.43 (0.089) |
| $\psi$(.), $p$(duration) | 3 | 82.57 | 8.51 | 0.01 | 0.18 (0.086) | 0.26 (0.118) |
| $\psi$(.), $p$(cloud cover) | 3 | 83.79 | 9.73 | 0.01 | 0.14 (0.053) | 0.44 (0.091) |
| $\psi$(level & south facing), $p$(.)[a] | 3 | 83.90 | 9.84 | 0.01 | 0.14 (0.056) | 0.43 (0.089) |
| $\psi$(level), $p$(.)[a] | 3 | 84.24 | 10.18 | 0.01 | 0.14 (0.053) | 0.43 (0.089) |
| $\psi$(south facing), $p$(.)[a] | 3 | 84.27 | 10.21 | 0.01 | 0.14 (0.053) | 0.43 (0.089) |
| $\psi$(area), $p$(.)[a] | 3 | 84.29 | 10.23 | 0.01 | 0.14 (0.053) | 0.43 (0.089) |
| $\psi$(.), $p$(refugia)[a] | 3 | 84.32 | 10.26 | 0.01 | 0.14 (0.053) | 0.43 (0.090) |
| $\psi$(connectivity), $p$(.) | 6 | 80.37 | 12.31 | 0.00 | 0.14 (0.098) | 0.43 (0.089) |
| $\psi$(.), $p$(survey-specific) | 7 | 81.91 | 15.85 | 0.00 | 0.14 (0.053) | 0.43 (0.195) |
| | | | | *Model averaged:* | 0.14 (0.048) | 0.42 (0.093) |
| | | | | | | |
| Adder | | | | | | |
| $\psi$(level & south facing), $p$(.) | 3 | 257.53 | 0.00 | 0.29 | 0.56 (0.072) | 0.57 (0.042) |
| $\psi$(.), $p$(.) | 2 | 261.51 | 1.98 | 0.11 | 0.56 (0.075) | 0.57 (0.042) |
| $\psi$(.), $p$(survey-specific) | 7 | 251.74 | 2.21 | 0.10 | 0.56 (0.075) | 0.57 (0.097) |
| $\psi$(level), $p$(.) | 3 | 259.82 | 2.29 | 0.09 | 0.56 (0.073) | 0.57 (0.042) |
| $\psi$(.), $p$(soil temp) | 3 | 260.36 | 2.83 | 0.07 | 0.56 (0.075) | 0.57 (0.042) |
| $\psi$(.), $p$(cloud cover) | 3 | 260.45 | 2.92 | 0.07 | 0.56 (0.075) | 0.58 (0.042) |
| $\psi$(.), $p$(duration) | 3 | 260.75 | 3.22 | 0.06 | 0.56 (0.075) | 0.56 (0.043) |
| $\psi$(area), $p$(.) | 3 | 260.80 | 3.27 | 0.06 | 0.56 (0.074) | 0.57 (0.042) |
| $\psi$(.), $p$(refugia) | 3 | 260.89 | 3.36 | 0.05 | 0.56 (0.075) | 0.57 (0.042) |
| $\psi$(.), $p$(air temp) | 3 | 260.92 | 3.39 | 0.05 | 0.56 (0.075) | 0.58 (0.042) |
| $\psi$(south facing), $p$(.)[a] | 3 | 261.50 | 3.97 | 0.04 | 0.56 (0.075) | 0.57 (0.042) |
| $\psi$(connectivity), $p$(.) | 6 | 257.74 | 6.21 | 0.01 | 0.56 (0.140) | 0.57 (0.042) |
| $\psi$(human impact), $p$(.) | 7 | 260.23 | 10.70 | 0.00 | 0.56 (0.075) | 0.57 (0.042) |
| | | | | *Model averaged:* | 0.56 (0.075) | 0.57 (0.048) |

| Model | $N$ | -2 log likelihood | Δ AIC | AIC Weight | $\hat{\psi}(s.e)$ | $\hat{p}(s.e)$ |
|---|---|---|---|---|---|---|
| Grass snake | | | | | | |
| $\psi$(.), $p$(soil temp) | 3 | 272.61 | 0.00 | 0.94 | 0.67 (0.075) | 0.43 (0.039) |
| $\psi$(.), $p$(survey-specific) | 7 | 270.12 | 5.51 | 0.06 | 0.66 (0.074) | 0.45 (0.087) |
| $\psi$(.), $p$(air temp) | 3 | 283.24 | 10.63 | 0.00 | 0.68 (0.076) | 0.43 (0.040) |
| $\psi$(.), $p$(.) | 2 | 291.38 | 16.77 | 0.00 | 0.67 (0.075) | 0.45 (0.041) |
| $\psi$(.), $p$(cloud cover) | 3 | 290.21 | 17.60 | 0.00 | 0.67 (0.075) | 0.44 (0.041) |
| $\psi$(.), $p$(duration)[a] | 3 | 291.23 | 18.62 | 0.00 | 0.67 (0.075) | 0.50 (0.043) |
| $\psi$(level), $p$(.)[a] | 3 | 291.34 | 18.73 | 0.00 | 0.67 (0.075) | 0.45 (0.041) |
| $\psi$(.), $p$(refugia)[a] | 3 | 291.35 | 18.74 | 0.00 | 0.67 (0.075) | 0.45 (0.041) |
| $\psi$(level & south facing), $p$(.)[a] | 3 | 291.36 | 18.75 | 0.00 | 0.67 (0.075) | 0.45 (0.041) |
| $\psi$(area), $p$(.)[a] | 3 | 291.37 | 18.76 | 0.00 | 0.67 (0.075) | 0.45 (0.041) |
| $\psi$(south facing), $p$(.)[a] | 3 | 291.37 | 18.76 | 0.00 | 0.67 (0.075) | 0.45 (0.041) |
| $\psi$(soil type), $p$(.) | 5 | 287.68 | 19.07 | 0.00 | 0.67 (0.123) | 0.45 (0.041) |
| $\psi$(.), $p$(experience) | 6 | 288.43 | 21.82 | 0.00 | 0.68 (0.077) | 0.44 (0.074) |
| $\psi$(connectivity), $p$(.) | 6 | 289.01 | 22.40 | 0.00 | 0.67 (0.140) | 0.45 (0.041) |
| | | | | *Model averaged:* | 0.67 (0.075) | 0.43 (0.042) |
| Smooth snake | | | | | | |
| $\psi$(.), $p$(soil temp) | 3 | 80.24 | 0.00 | 0.88 | 0.16 (0.054) | 0.64 (0.059) |
| $\psi$(.), $p$(survey-specific) | 7 | 76.66 | 4.42 | 0.10 | 0.16 (0.054) | 0.62 (0.133) |
| $\psi$(area), $p$(.) | 3 | 90.11 | 9.87 | 0.01 | 0.16 (0.050) | 0.62 (0.076) |
| $\psi$(.), $p$(refugia) | 3 | 90.94 | 10.70 | 0.00 | 0.16 (0.054) | 0.67 (0.071) |
| $\psi$(level & south facing), $p$(.) | 3 | 91.90 | 11.66 | 0.00 | 0.16 (0.052) | 0.62 (0.076) |
| $\psi$(.), $p$(.) | 2 | 94.65 | 12.41 | 0.00 | 0.16 (0.075) | 0.62 (0.076) |
| $\psi$(), $p$(air temp) | 3 | 93.44 | 13.20 | 0.00 | 0.16 (0.055) | 0.59 (0.082) |
| $\psi$(.), $p$(duration) | 3 | 93.80 | 13.56 | 0.00 | 0.16 (0.054) | 0.68 (0.092) |
| $\psi$(south facing), $p$(.) | 3 | 94.08 | 13.84 | 0.00 | 0.16 (0.054) | 0.62 (0.076) |
| $\psi$(level), $p$(.)[a] | 3 | 94.26 | 14.02 | 0.00 | 0.16 (0.054) | 0.63 (0.076) |
| $\psi$(.), $p$(cloud cover)[a] | 3 | 94.58 | 14.34 | 0.00 | 0.16 (0.054) | 0.62 (0.076) |
| $\psi$(connectivity), $p$(.) | 6 | 90.36 | 16.12 | 0.00 | 0.16 (0.100) | 0.62 (0.076) |
| | | | | *Model averaged:* | 0.16 (0.054) | 0.64 (0.067) |

$N$ = Number of parameters in the model, $\hat{\psi}$ = estimated occupancy probability, and $\hat{p}$ = estimated detection probability. Models that failed to converge were discarded. 15 models were therefore run for each species in this data set. Model notation used is:

| | | | |
|---|---|---|---|
| (.) | The parameter was a constant | (air temp) | Mean air temperature during surveys |
| (survey-specific) | The parameter varied over time | (soil temp) | Mean soil temperature during surveys |
| (area) | The area of the site measured in hectares | (cloud cover) | Proportion of the sky covered by cloud |
| (connectivity) | The connectivity of the site with other areas of habitat | (duration) | Length of the survey in minutes |
| (human impact) | The amount of human activity at the site | (refugia) | The total number of refugia checked |
| (level) | The proportion of the site that was level | (experience) | The field experience of each surveyor |
| (south facing) | The proportion of the site that faced south | | |
| (level & south facing) | The proportions of the previous two categories combined | | |
| (soil type) | Classification of site soil types into acid, alkaline or neutral | | |

Further details of covariates are given in the text.

[a] note that in these models the covariate can be considered to be acting as a “pretending variable” [30]
