# Supplementary material for: When Is a Species Declining? Optimizing Survey Effort to Detect Population Changes in Reptiles
Source: PLoS One. 2012 Aug 22;7(8):e43387. doi: 10.1371/journal.pone.0043387 (PMC3425567; doi:10.1371/journal.pone.0043387)
Supplement: Table S6 — Monthly variation in detection probability. (PDF) [file pone.0043387.s009.pdf]

Table S6: Monthly variation in detection probability (s.e.) by species and data set

Species	March	April	May	June	July	September
Slow-worm						
2010, 29 sites	0.44(0.096)	0.97(0.034)	0.90(0.057)	0.83(0.070)	0.79(0.075)	0.97(0.034)
2010, 45 sites	0.49(0.082)	0.92(0.043)	0.92(0.044)	0.82(0.061)	0.82(0.061)	0.97(0.025)

Only includes the single species where a model suggesting variation in detection over time in Tables S3, S4 and S5 accounted for >0.5 of AIC weight.
