# Supplementary material for: When Is a Species Declining? Optimizing Survey Effort to Detect Population Changes in Reptiles
Source: PLoS One. 2012 Aug 22;7(8):e43387. doi: 10.1371/journal.pone.0043387 (PMC3425567; doi:10.1371/journal.pone.0043387)
Supplement: Table S7 — Power simulation results. (DOCX) [file pone.0043387.s010.docx]

**Table S7: Actual power achieved with the designs indicated in Figure 1 based on 5000 simulations.**

| species | R | K |  |  | power |  |  |
| --- | --- | --- | --- | --- | --- | --- | --- |
|  |  |  | 0.75 | 0.80 | 0.85 | 0.90 | 0.95 |
| adder | 0.10 | 3 | 0.75 | 0.80 | 0.84 | 0.91 | 0.95 |
|  |  | 4 | 0.75 | 0.80 | 0.85 | 0.91 | 0.95 |
|  | 0.15 | 3 | 0.74 | 0.80 | 0.84 | 0.90 | 0.95 |
|  |  | 4 | 0.75 | 0.80 | 0.85 | 0.90 | 0.95 |
|  | 0.30 | 3 | 0.74 | 0.79 | 0.84 | 0.89 | 0.94 |
|  |  | 4 | 0.75 | 0.80 | 0.84 | 0.89 | 0.95 |
| grass snake | 0.10 | 3 | 0.75 | 0.80 | 0.85 | 0.90 | 0.95 |
|  |  | 4 | 0.75 | 0.80 | 0.85 | 0.90 | 0.95 |
|  | 0.15 | 3 | 0.75 | 0.80 | 0.84 | 0.90 | 0.95 |
|  |  | 4 | 0.76 | 0.80 | 0.86 | 0.90 | 0.95 |
|  | 0.30 | 3 | 0.75 | 0.80 | 0.84 | 0.90 | 0.94 |
|  |  | 4 | 0.75 | 0.80 | 0.85 | 0.90 | 0.95 |

Figures given are for the most demanding species (grass snake and adder) at K =3 and K = 4 where K = number of replicates (surveys) per site.
